# Retrospective analysis of secondary resection of the cervical stump after subtotal hysterectomy: why and when?

**DOI:** 10.1007/s00404-021-06193-6

**Published:** 2021-08-28

**Authors:** Felix Neis, Christl Reisenauer, Bernhard Kraemer, Philipp Wagner, Sara Brucker

**Affiliations:** Department of Obstetrics and Gynecology, University Hospital Tübingen, Calwerstrasse 7, 72076 Tübingen, Germany

**Keywords:** Subtotal hysterectomy, Supracervical hysterectomy, Resection of the cervix, Cervical stump resection, Secondary resection of the cervical stump

## Abstract

**Purpose:**

The rates of hysterectomy are falling worldwide, and the surgical approach is undergoing a major change. To avoid abdominal hysterectomy, a minimally invasive approach has been implemented. Due to the increasing rates of subtotal hysterectomy, we are faced with the following questions: how often does the cervical stump have to be removed secondarily, and what are the indications?

**Methods:**

This was a retrospective, single-centre analysis of secondary resection of the cervical stump conducted from 2004 to 2018.

**Results:**

Secondary resection of the cervical stump was performed in 137 women. Seventy-four percent of the previous subtotal hysterectomy procedures were performed in our hospital, and 26% were performed in an external hospital. During the study period, 5209 subtotal hysterectomy procedures were performed at our hospital. The three main indications for secondary resection of the cervical stump were prolapse (31.4%), spotting (19.0%) and cervical dysplasia (18.2%). Unexpected histological findings (premalignant and malignant) after subtotal hysterectomy resulted in immediate (median time, 1 month) secondary resection of the cervical stump in 11 cases. In four patients, the indication was a secondary malignant gynaecological disease that occurred more than 5 years after subtotal hysterectomy. The median time between subtotal hysterectomy and secondary resection of the cervical stump was 40 months. Secondary resection of the cervical stump was performed vaginally in 75.2% of cases, laparoscopically in 20.4% of cases and abdominally in 4.4% of cases. The overall complication rate was 5%.

**Conclusion:**

Secondary resection of the cervical stump is a rare surgery with a low complication rate and can be performed via the vaginal or laparoscopic approach in most cases. The most common indications are prolapse, spotting and cervical dysplasia. If a secondary resection of the cervical stump is necessary due to symptoms, 66.6% will be performed within the first 6 years after subtotal hysterectomy.

## Introduction

Hysterectomy is one of the most common gynecological surgeries. As a result of uterus-preserving techniques, the number of hysterectomies worldwide is decreasing [1]. Due to the advantages of minimally invasive surgery and technological development, the vaginal and laparoscopic approaches to hysterectomy have increasingly replaced abdominal hysterectomy. Numerous international guidelines advise the use of either a vaginal or laparoscopic approach for hysterectomy for benign indications [2, 3]. To date, the available literature has not established any type of hysterectomy as being the safest method. Nevertheless, the vaginal approach is the one that is the most minimally invasive because it involves the fewest incisions. However, laparoscopic approaches for total laparoscopic hysterectomy (TLH) and laparoscopic subtotal hysterectomy (LASH) are becoming more relevant due to their low complication rates and short lengths of hospital stay [2, 3].

Despite the FDA warning against power morcellation in 2014 [4], the number of subtotal hysterectomy (SH) procedures in Germany is still increasing [5]. According to the recommendations of the German Society of Gynecology and Obstetrics, patients without suspicion of a malignant uterine tumour might be offered LASH after extensive counselling about the potential risk of tumour cell dissemination when an occult malignant disease is present and power morcellation is used. In December 2020 a FDA guidance described post-menopausal women or those over 50 years of age, as well as those where an en bloc removal through the vagina or via a mini-laparotomy is possible as contraindications for laparoscopic power morcellation [6]. If a laparoscopic power morcellator is applied a containment system compatible with the laparoscopic power morcellator should be used [6]. Consequently, patients have to be informed about alternatives to LASH [7].

Main difference between TLH and LASH is the risk of persisting vaginal bleeding from the cervical stump in approximately 11–19% and higher rates of urinary incontinence (RR 1.37) in case of a LASH after a 5 year follow up [8, 9], as well as the persisting risk of dysplasia of the cervix and cervical cancer. In contrast, patients who undergo LASH show a faster resumption of normal daily activities and resume sexual activities sooner than those undergoing TLH [10], which is of special interest in young and active women.

In Germany, the patient is referred to the hospital with the indication for hysterectomy by resident gynecologists. The reasons for indicating SH are, especially in middle aged and sexual active women, the unaffected anatomy of the vagina as well as the enduring possibility of performing a cervical cytology for cancer screening, which secures the resident gynecologist a constant (1–3 years interval) routine checkup of these patients. Suspicious pap smears in the patient’s history, a sonographic suspicious endometrium or myometrium are absolute contraindications for SH procedures.

Resection of the cervix after laparoscopic or abdominal SH is performed rarely, and there is no report addressing the indications or frequency of secondary resection of the cervical stump (SRC) after SH. Before the FDA warning against power morcellation SH was a common approach to hysterectomy worldwide [11–14]. Thus, the aim of this study was to clarify the background of SRC in a large population-based, single-centre cohort.

## Material and methods

We performed a retrospective analysis of all patients receiving SRC after SH at the Department of Women’s Health at the University Hospital in Tübingen, Germany, from January 2004 to December 2018.

According to the surgery procedure codes (5-684 and 5-673), the resections and amputations of the cervix were extracted from the digital patient file (SAP^®^ clinical documentation system), and relevant data were collected and transferred to an EXCEL file. The following data were recorded: dates of SH and SRC, patient characteristics (age, BMI, previous surgeries, parity, ASA score), indications for SH and SRC, time of surgery, duration of hospital stay, postoperative histological findings and complications. Complications were classified according to Clavien-Dindo [15]. Follow up was conducted by reviewing the digital patient file.

The study was approved by the Ethics Commission at the Faculty of Medicine and the University Hospital in Tübingen (Number: 588/2018BO2, 25th of May 2019).

## Results

According to surgery procedure codes, all resections of the cervix from January 2004 to September 2018 were analysed. In total, 498 resections of the cervix were performed in our unit; 364 patients were excluded because the surgery procedure code was also used in cases of resection/amputation of the cervix during vaginal morcellation and in cases of trachelectomy for early-stage cervical cancer. Thus, 137 patients with SRC after SH were included in the study (27.5%). Between 2004 and 2018, 5209 SHs were performed at our hospital.

A total of 102 (74.5%) patients with indications for SRC had SH in our hospital, and 35 (25.5%) were referred to our hospital after a previous SH at another hospital. Of these 137 SH procedures, 89.1% were performed via the laparoscopic route, 8.0% via the abdominal route by a horizontal incision and 2.9% via the abdominal route by a vertical incision. The main indications for SH were fibroids (64.8%), bleeding disorders (15.9%), endometriosis (9.7%), tumour of the ovary (4.1%), pelvic organ prolapse (POP) (3.4%), and pain that was not related to fibroids or endometriosis (1.4%); in one patient, SH was performed at her request (0.7%). In six cases of symptomatic POP, mesh-supported cervico-sacropexy (CSP) was performed at the same time as SH. None of the remaining patients had symptomatic POP before SH.

The average age of our patients was 52.3 years (range 26–83 years). Twenty-one patients (15.3%) were nulliparous, and 108 patients (78.8%) were primiparous or multiparous. Among eight patients (5.8%), there were no available data on parity. The majority of our patients were healthy: 23.4% had ASA scores of 1, 72.2% had ASA scores of 2 and 4.4% had ASA scores of 3.

The median time between SH and SRC was 40 months (range 0–647 months). Among cases in which SRC was performed after SH for reasons other than a premalignant or malignant disease, the cumulative risk for postoperative SRC was 9.5% in the first 6 months, 16.6% in the first year, 46.8% after 3 years, 66.6% after 5 years and 87.5% after 10 years (Table 1). If there was an unexpected histological finding after SH, SRC was performed at a median of 1 month (range 0–4 months) after SH.

**Table 1:** Time between subtotal hysterectomy (SH) and secondary resection of the cervical stump (SRC) if there was no unexpected histological finding (*n* = 126 of 137)

Time till SRC (months)	Frequency (*n*)	Percent (%)	Cumulative percent (%)
0–6	12	9.5	9.5
7–12	9	7.1	16.6
13–24	15	11.9%	28.5
25–36	23	18.3	46.8
37–60	25	19.8	66.6
61–120	24	19.1	85.7
121 +	18	14.3	100.0

If an unexpected histological finding was diagnosed, the median time until surgery was 1 month (range 0–4)

Between 2004 and 2011, there were 1–9 SRCs per year. In the years following 2011, the number of SRCs increased until 2015, when 21 SRCs per year were performed. From 2015 to 2018, the number of SRCs decreased in a stepwise manner (2018: 11 SRC/year).

The approach to SRC was vaginal in 75.2% of cases, laparoscopic in 20.4% of cases, and abdominal in 4.4% of cases. There was one conversion from laparoscopy to laparotomy (0.7%) because of extensive parasitic fibroids after LASH with morcellation. The average time of surgery for all indications was 86.2 min (range 20–510 min); for benign conditions, the average time of surgery was 78.7 min (range 20–337 min). In 102 SRCs (74.5%), additional interventions, such as colporrhaphy, adhesiolysis, resection of ovarian cysts, and resection of endometriosis or fibroids, were performed simultaneously.

Indications for SRC are presented in Table 2. The main indications for SRC were symptomatic POP (31.4%), spotting (19.0%), dysplasia and a suspicious Pap smear of the cervix (18.2%). The other indications, which had lower rates, were pain, fibroids, endometriosis, cancer that was incidentally detected immediately after the initial SH, adhesions, premalignant lesions of the ovary or fallopian tube, hyperplasia of the endometrium, cancer more than 5 years after SH, vesicovaginal fistula, patient request and cysts of the cervix. In 25 cases, there was more than one indication for SRC.

**Table 2:** Indications for secondary resection of the cervix (SRC, %)

Indication	*N*	Percent
Prolapse	43	31.4
Spotting	26	19.0
Dysplasia/suspicious Pap smear	25	18.2
Cancer incidentally detected by LASH	10	7.3
Endometriosis	7	5.1
Pain	6	4.4
Fibroids	5	3.7
Hyperplasia of the endometrium	4	2.9
Cancer after more than 5 years	4	2.9
Borderline lesion of the ovary/STIC incidentally detected by LASH	3	2.2
Cervical cyst	2	1.5
Fistula	1	0.7
Wish of the patient	1	0.7
Total	137	100.0

The average age of the patients for the three most common indications was 58.0 years for cases of symptomatic POP, 47.4 years for patients with spotting and 50.9 years for cases of dysplasia. The average time between SH and SRC was only 34.4 months for cases of spotting, 60.6 months for cases of dysplasia and 113.5 months for cases of symptomatic POP.

Between 2004 and 2018, a total of 5209 SH procedures were performed in our hospital. Of these cases, 11 had unexpected histological findings (0.21%) during SH that required subsequent SRC. These findings were premalignant lesions in 3 (0.06%) cases (two borderline lesions of the ovary, one serous tubal intraepithelial carcinoma (STIC)) and unexpected early-stage malignant diseases in 8 (0.15%) cases (four endometrial cancers, two sarcomas and two ovarian cancers). Two SRCs were performed due to unexpected findings of malignancy after SH in external hospitals (one sarcoma and one endometrial cancer) (Table 3).

**Table 3:** Total number of subtotal hysterectomy (SH) procedures and all hysterectomy (HE) procedures in Tübingen per year

	Year									
	2010	2011	2012	2013	2014	2015	2016	2017	2018	Total 2004–2018
Total number of SHs in Tübingen	417	465	471	452	487	452	390	407	437	5209
Total number of HEs in Tübingen	889	893	1001	885	955	965	868	834	829	10,820
Unexpected histological findings	1 (0.24%)	0	0	2 (0.44%)	2 (0.41%)	0	1 (0.26%)	4 (0.98%)	1 (0.23%)	11 (0.21%)
Unexpected malignancy	1 (0.24%)	0	0	0	2 (0.41%)	0	1 (0.26%)	3 (0.74%)	1 (0.23%)	8 (0.15%)
Endometrial cancer	0	0	0	0	2 (0.41%)	0	1 (0.26%)	1 (0.25%)	0	4 (0.08%)
Sarcoma	1 (0.24%)	0	0	0	0	0	0	1 (0.25%)	0	2 (0.04%)

Distribution of unexpected histological findings per year, percentage in clamps, and unexpected histological findings per total number of SHs per year. From 2004 to 2009, there were no unexpected histological findings

Two additional malignancies were diagnosed in an external hospital (one endometrial cancer and one sarcoma)

After a minimum of 66 months after SH, four patients had malignant disease requiring SRC (two ovarian cancers, one cervical cancer, one vaginal cancer). This corresponds to 0.077% (4/5209) of the total number of SHs performed during the time period of this analysis. All patients who underwent SH procedures at our hospital had nonsuspicious Pap smears of the cervix within 12 months prior to hysterectomy; however, we were not able to determine the preoperative cervical Pap smears of the patients who underwent SH at an external hospital.

There were 7 grade I–V complications (5.11%) per the Clavien-Dindo classification [15] (Table 4). Of these seven cases, there were six cases (4.38%) with severe complications, namely, Clavien-Dindo III–V complications, that occurred during SRC.

**Table 4:** Intra- and postoperative complications according to the Clavien-Dindo classification [15]

	*N* (%)
**Intraoperative complications**	
Lesion of the bladder	1 (0.73%)
**Postoperative complications (Clavien-Dindo I + II)**	
Haematoma treated conservatively (day 3)	1 (0.73%)
**Postoperative complications (Clavien-Dindo III–V)**	
Bleeding (2 × day 1, 1 × day 7, 1 × day 28)	4 (2.9%)
Voiding difficulties after anterior colporrhaphy (day 7)	1 (0.73%)
Infected haematoma (day 8)	1 (0.73%)
Total number of peri- and postoperative Clavien-Dindo III–V complications	6 (4.38%)
Total number of peri- and postoperative Clavien-Dindo I–V complications	7 (5.11%)

## Discussion

In Germany, the LASH procedure is a common surgical approach for hysterectomy [5]. Although the frequency of hysterectomy is decreasing, that of SH is increasing [16]. The German guidelines for hysterectomy for benign indications advise the vaginal approach as the first-choice surgical approach [2]. If a vaginal hysterectomy is not possible, hysterectomy by laparoscopy should be used. These guidelines state that there is no evidence that any laparoscopic hysterectomy procedure is superior to the other [2]. Other international guidelines state the same thing [3, 17, 18].

Dysmenorrhoea and hypermenorrhoea caused by fibroids or adenomyosis uteri are the main indications for LASH. Patients undergoing LASH for benign indications show faster recovery in daily life and sexual function than those undergoing TLH [19]. Andersen analysed the difference of TLH and LASH after a follow-up of 5 years. The main difference that could be demonstrated in his article was the risk of persisting vaginal bleeding from the cervical stump in approximately 11% and a trend towards higher rates of urinary incontinence (RR 1.37, *p* = 0.052) in case of a LASH after a 5 year follow up, which was not significant [8]. Borendal showed in a Swedish retrospective register study a rate of persisting vaginal bleeding of 18.6% after SH with and without cervical treatment in a follow-up time of one year [9]. Still 90% of these women were satisfied with the surgery, although women with vaginal bleeding were less confident.

Nevertheless, SRC becomes necessary. To our knowledge, this is the first study to analyse the indications and time frame of SRC after SH.

The three main indications for SRC in our study were symptomatic POP, spotting and dysplasia of the cervix.

In total, 31.4% of all SRCs were performed because of symptomatic POP. Patients with symptomatic POP were, as is typical, older than the patients with dysplasia and spotting. The average time between the SH and SRC in the case of prolapse was the longest, at 113 months. There were only six patients who needed prolapse treatment at the same time as SH and received CSP simultaneously. The indications for these 6 SRCs were recurrent symptomatic POP in three cases, one case of infection of the mesh with recurrent symptomatic POP, one vesicovaginal fistula and pain caused by the tension of the mesh in one case. Five of these 6 SHs with CSP were performed at an external hospital. There average time to SRC after SH with CSP was 61 months. This might suggest that patients who need treatment because of POP prior to SH are at a higher risk of relapse surgery. In his systematic review, Maher showed a risk of repeat prolapse surgery after CSP of 4% [20]. Because we perform more than 33 CSPs per year in our hospital [21], the risk of repeat prolapse surgery is below 1%.

In a Cochrane review, Lethaby et al. showed no difference between subtotal and total hysterectomy but a significantly higher risk of ongoing menstrual bleeding (OR 19.0) after SH [22]. Schmid et al. showed that the incidence of spotting is 10.7% and that the rate of spotting could be reduced by excision of the endocervix to 1.4% [23]. Cooper presented a higher percentage (19%) of persistent bleeding after LASH [14]. In our hospital, coagulation of the cervical channel is a standard procedure during LASH before peritonealization of the cervical stump. In our cohort, the indication for SRC due to spotting was 18%. In the time frame between 2004 and 2018, we performed 5209 SHs, which led to only 26 SRCs for spotting after LASH (0.5%). Although spotting was the second most common indication for SRC in our cohort, it can be assumed that the majority of women with spotting have such a low amount of bleeding or are not bothered by the spotting so that no further surgery is necessary. The patients in our cohort who needed reintervention due to spotting obtained their SRCs after a median of 34 months following SH. It can therefore be assumed that if patients are affected by bothersome spotting, the majority will receive a reoperation within the first 3 years after SH.

The third most common indication for SRC in our cohort was dysplasia of the cervix (17%). All patients who received LASH in our centre had an unsuspicious cervical Pap smear at least one year before surgery. This fact shows the importance of cervical cancer screening after SH. As dysplasia of the cervix is highly associated with human papilloma virus (HPV), the newly implemented screening programme in Germany, with the combination of cytology and HPV testing [24], might change the proportion of SRCs with dysplasia of the cervix in the future. A recent suspicious Pap smear and a previous intervention at the cervix due to dysplasia or other pathologies of the cervix are currently contraindications for SH. If we know that our patient has a positive HPV status, we should minimize the risk of SRC by not offering SH.

Eleven SRCs were performed because of unexpected histological findings after SH. Nine of these were hysterectomies that were performed in our centre, and two were performed in external hospitals. During the study period (2004–2018), 5209 SHs were performed in our centre. In this cohort, three unexpected premalignant lesions and eight unexpected malignant lesions were identified (0.06 and 0.15%) after SH. All were early-stage malignancies, with a favourable prognosis. In a previous study, our research group analysed the risk of a uterine malignancy (sarcoma and endometrial cancer) at our university hospital after hysterectomy for benign indications and found that the risk of unexpected malignancies of the uterine corpus after LASH was 0.21% [25]. Consistent with this study, our data show that the risk of unexpected malignancies seems to be very low. The incidence of unexpected uterine malignancies has a wide range from 0.2 to 1.24% as these are often data from small cohorts and retrospective single centre analysis [26–29]. In 2020 Desai published data prior to the FDA warning (2003–2013) describing a proportion of 0.22% sarcoma and 0.75% endometrial cancer in a regional databased survey of 229,536 hysterectomies in New York [30]. In total occult uterine malignancies were found in this population is 0.96%. Compared to these studies the incidence of uterine malignancies in our cohort is in the lower range. This might be a result of our stringent indication, as any suspicious sonographic finding or medical history is a contra indication for LASH.

After a minimum of 66 months (5.5 years) after SH, we found four malignant diseases requiring SRC. The two ovarian cancers were not related to the method of hysterectomy. The malignancies of the two patients with HPV-related cancers of the cervix and the vagina might have been prevented if the HPV status of each patient had been known.

The rates of complications related to hysterectomy differ among the methods of access. The FINHYST trial demonstrated major complication rates ranging from 2.6 to 4.3% and total complication rates ranging from 11.7 to 19.2% among vaginal, laparoscopic and abdominal hysterectomy [31]. In our cohort, complications occurred in 5.11% of cases, and major complications classified as Clavien-Dindo III–V occurred in 4.38% of cases. The rates of total complications were equivalent to or lower than those in other recent studies [32–34]. The low rate of minor complications in contrast to other studies might be a result of the retrospective design of our study.

Between 2005 and 2018 in Germany, 1,766,865 hysterectomies (procedure codes 5-682 and 5-683) were performed, and 244,804 of these were SHs (procedure code 5-682) (16.1%) [35]. Only in 10,785 cases was the procedure code for resection (procedure code 5-684) or amputation of the cervix (procedure code 5-673) noticed (4.4%) [16]. In our cohort, these procedure codes (procedure codes 5-673 and 5-684) described only 27.5% of the SRC procedures. The other cases were resections of the cervix during vaginal hysterectomies with morcellation, trachelectomy or large conization for early-stage cervical cancer and were excluded from the analysis. If we update the German data to reflect the fact that only approximately one-quarter of the OPS codes fit the surgical approach of SCR in our cohort, the data would show that only 1.2% of all SHs in Germany will need an SRC in the future.

The limiting factors of our study are its retrospective study design and single-centre analysis. As there was no written follow-up, only the complications which were treated in our hospital were analysed in this retrospective study. Despite the huge catchment area of our hospital the resident gynaecologists send their patient, in case of a complications, back to the hospital where the surgery was performed. Nevertheless, this limits the significance of the complications analysis.

However, to our knowledge, this is the first analysis of SRC.

Even so, due to the low number of cases per year and the long timeframe between SH and SRC, prospective studies will be difficult to carry out.

## Conclusion

The SRC is a rare surgery after SH. In most cases, minimally invasive access to SRC is possible. Symptomatic POP, spotting and dysplasia of the cervix are the main indications. The timeframe from SH to SRC, which ranges from 34 to 113 months, depends on the indication for SRC. If a SH is indicated, the number of SRCs might be reducible if we explain the risk of spotting after SH more extensively to the patients. If we add a positive high-risk HPV status as a contraindication for SH, the rate of SRC might be reduced even more. The complication rate of SRC is comparable to those of vaginal and laparoscopic hysterectomy. The patient, suitable for SH, needs to be well screened and extensively informed about the risk of detecting a malignant or premalignant disease incidentally during SH.

## Data Availability

All data generated or analyzed during this study are included in this published article.

## Abbreviations

ASA score: American Society of Anesthesiologists score
CSP: Cervico-sacropexy
HPV: Human papilloma virus
LASH: Laparoscopic subtotal hysterectomy
POP: Pelvic organ prolapse
SH: Subtotal hysterectomy
SRC: Secondary resection of the cervical stump
